# Competing for the same value segments? Insight into the volatile Dutch political landscape

**DOI:** 10.1371/journal.pone.0190598

**Published:** 2018-01-11

**Authors:** Hester van Herk, Pieter C. Schoonees, Patrick J. F. Groenen, Joost van Rosmalen

**Affiliations:** 1 Department of Marketing, Vrije Universiteit, Amsterdam, the Netherlands; 2 Department of Marketing Management, Rotterdam School of Management, Erasmus University, Rotterdam, the Netherlands; 3 Econometric Institute, Erasmus University Rotterdam, Rotterdam, the Netherlands; 4 Department of Biostatistics, Erasmus MC, Rotterdam, the Netherlands; University of Vermont, UNITED STATES

## Abstract

Values are central to public debates today. Human values convey broad goals that serve as guiding principles in a person’s life and value priorities differ across people in society. Groups in society holding opposing values (e.g., universalism versus security) will make different choices when voting in an election. Whereas over time, values are relatively stable, the number and type of political parties as well as the political values they communicate and disseminate have been changing. Groups of people holding the same human values may therefore vote for another (new) party in a later election. We focus on analyzing the relationship between human values and voting in elections, introducing a new methodology to analyze how value profiles relate to political support over time. We investigate the Dutch multi-party political system over five waves of the European Social Survey, spanning 2002 until 2010. Whilst previous research has focused on individual values separately and focused on voters only, we (1) distinguish groups holding a similar set of opposing and compatible values (value profile) instead of focusing on single values in the the entire population; (2) incorporate a correction for differences in scale use in our model; (3) compare voting over time; (4) include non-voters, a growing group in Dutch society. We find evidence that specific value profiles are related to voting for a specific set of political parties. We also find that specific value profiles distinguish non-voters from voters and that voters for populist parties resemble non-voters.

## Introduction

Recent years have seen substantial changes to political landscapes in many countries [1]. Major established political parties have lost ground to new parties, most notably the populist parties. Concurrently, both the proportion of the electorate choosing to abstain from voting and the proportion switching between parties are on the rise in many countries [2]. One possible reason for these changes in voting behavior is that the basic human values parties emphasize have become in-congruent with the values people hold. We investigate this idea in this paper.

Human values refer to what people hold important in life, for example, freedom, security or power. They motivate what people do and help them to select or justify their behavior [3]. For instance, people valuing security will more likely be motivated to vote for a party that promises to ensure their safety (through security values). Moreover, people valuing universalism will likely be more motivated to vote for a party recognising the positive aspects of immigration and understanding others (appealing at universalism values). When voting for a party reflecting their own human values, people will feel they are behaving in line with their own values. Moreover, human values are more stable over time than political values [4] and they enable predicting political choice [5].

We use the currently dominant theory of human values by Schwartz [3, 6]. Schwartz proposed a nearly universal structure of 10 human values, which has been validated in over 75 nations worldwide, using various measurement instruments [7]. The theory has been used to assess differences in various behaviors including voting behaviour [8]. In the studies on voting behavior, researchers typically took the political parties or political left-right orientation as a starting point and investigated whether and which specific values differed between parties or orientations. In this way, it is possible to determine which values distinguish one party from another; however, it is not possible to assess which parties voters may choose given their own value profile on the complete set of 10 values.

In the current study we fill this gap, as we start with determining groups (segments) of people having similar profiles on all 10 values. This builds upon the recent finding that the theoretical (circular) structure of values also holds in general within individuals [9, 10], giving each individual a value profile in which the compatibilities and oppositions between the 10 values can be distinguished. This will be explained further in the theory section. Combined with the notion that people differ with respect to the specific value(s) they consider important and unimportant, we will determine whether distinguishable groups of people with the same value profiles exist in the population.

We also address a main measurement issue in values research, which is that the absolute scores on a specific value do not matter. Rather, it is the relative score in comparison with other values that matters. For instance, it is not the absolute importance a person attaches to the value security, it is the relative importance of security to other values, such as universalism, that matters. Furthermore, as rating scales are used, measures will be contaminated with response styles [11–13]. Response styles are a general tendency to use rating scale categories independent of item content [14]. For instance, some respondents will mainly tick the “most important” category, whereas others with the same value priorities will mainly tick the “important” category. Given the differences in scale use, in values measurement, a correction for response style is considered essential [15]. As we focus on groups of respondents (segments), we employ the LC-BML model [16]; this model enables us to determine value segments corrected for differences in response styles. Moreover, this model enables correcting for different kinds of response styles, not only the common ones such as extreme response style or acquiescence.

Summarizing, in contrast to other studies that focused on how preferences for political parties correlated with specific values [5, 17, 18], we look from the perspective of the individual and focus on how their complete value profiles relate to voting for (homogeneous groups of) political parties or to abstain from voting. In particular, our contributions are: (1) Assessing how many value segments can be distinguished in the population; (2) Positing how these segments relate to voting for specific sets of political parties; (3) Showing how value segments relate to voting over time, taking into account that the parties participating in the elections may change, and that people may abstain from voting; and (4) Assessing whether voting for political parties can be predicted using value segments.

We illustrate our approach by using data from the Netherlands, a nation with a multi-party system where many new parties enter the political arena, such as in the recent 2017 elections. Moreover, compared to other nations, the Netherlands is a nation in which electoral volatility is very high [19] [20] making the Netherlands a suitable setting for our study.

In the next section, we briefly review the theory of human values.

### Human values

Human values convey broad goals that serve as guiding principles in a person’s life for the selection of behaviours and the evaluation of behaviours and events [3]. In the current dominant theory of individual values [3], Schwartz distinguishes 10 distinct values, benevolence, universalism, self-direction, stimulation, hedonism, achievement, power, security, conformity and tradition.

Benevolence refers to protecting the well-being of people with whom one has frequent personal contact. Universalism refers to understanding, tolerating and protecting all people, including those far away and it also includes taking care of nature and the natural environment. Self-direction entails valuing independent thought, opting for action, and being creative and investigative. Stimulation refers to valuing exciting, new and challenging things. Achievement refers to showing personal success by demonstrating competences according to social standards. Power refers to valuing social status and prestige by having control over people and resources. Security refers to safety, harmony, and stability of society, of relationships, and of self. It entails two types of security; one that focuses primarily on individual interests (e.g., avoiding danger, living in a secure environment), the other focuses wider group interests (e.g., having a strong government). Conformity refers to restraint of actions, tendencies, and impulses that can upset or harm others and violate social expectations or norms. Finally, tradition refers to valuing respect for, involvement in and acceptance of ideas that traditional culture or religion requires from people.

**Fig 1:**
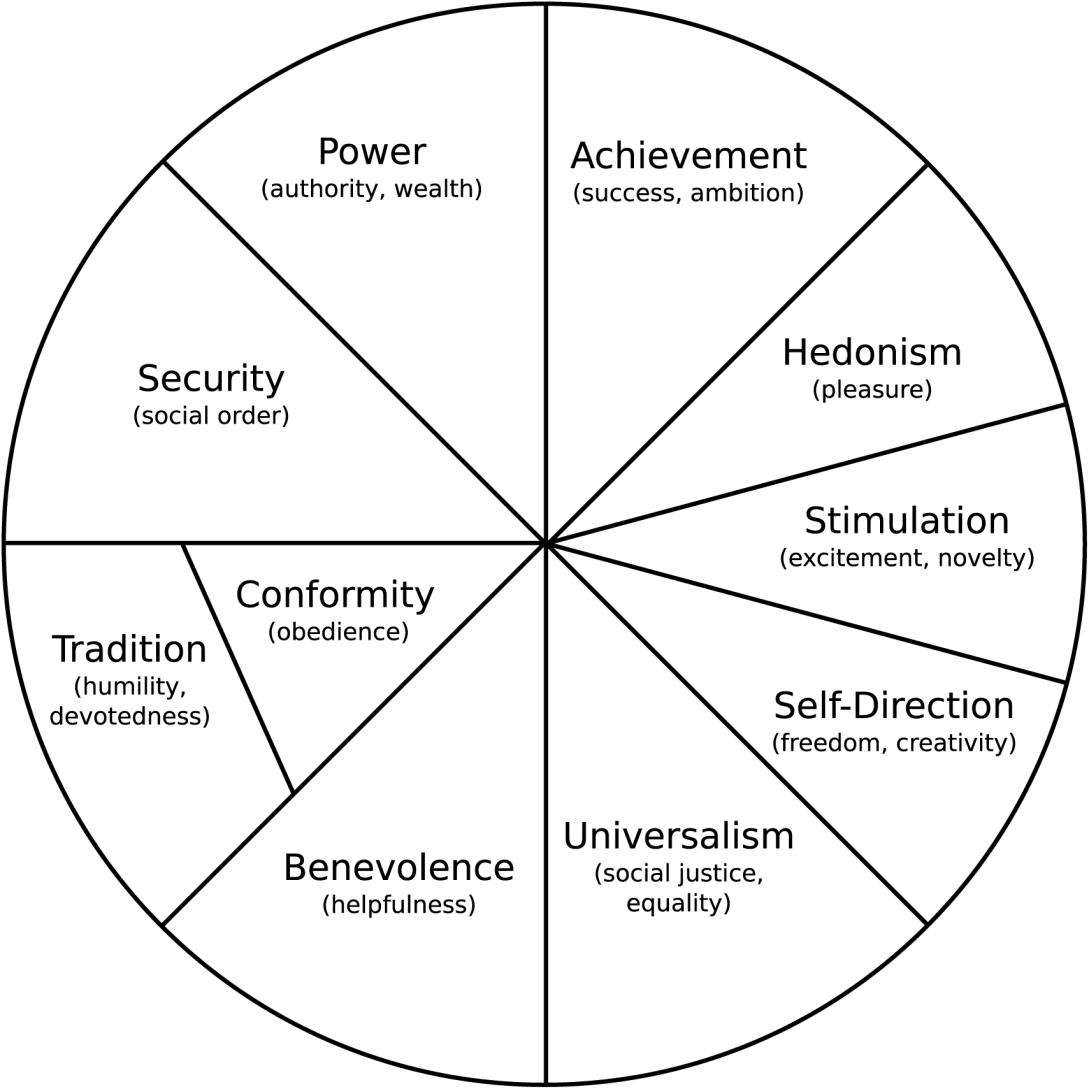
Schwartz’s value circumplex. Schwartz’s value circumplex, showing the relationship between human values.

These 10 values are organized in a circular structure that represents the compatible and opposing relationships between the values. Here we briefly discuss this structure, as can be seen in Fig 1. Relationships between values such as benevolence and universalism or between benevolence and tradition are positive and result in positions adjacent to each other on the circumplex. Benevolence and universalism share the acceptance of others and having concern for their welfare and benevolence and tradition share the importance of involvement in the in-group. The opposing power and benevolence do not coincide as valuing “dominance over others” (power) conflicts with valuing “protecting the well-being of people with whom one has frequent personal contact” (benevolence); the conflicting perspectives result in a position on opposite sides of the circumplex.

Studies on human values have long focused on the structure of values across all individuals in a sample. Recent research [10] shows that the circular structure of values not only exists across individuals, but also tends to hold on an individual basis. Suppose the structure in Fig 1 holds for a particular individual. If she considers benevolence to be of the highest importance, then she will consider the value power to be the least important. Based on this choice of benevolence as the most important, the individual will either consider tradition or universalism as the second most important value. This follows from the adjacency of these values to benevolence in Fig 1. Suppose she prefers universalism over tradition, then the theory predicts that she will also consider self-direction important, and security and conformity less important.

As people differ in the importance they attach to the different values, we expect that there are distinct groups of people sharing the same value priorities. For instance, there might not only be a group considering security values of highest importance and considering stimulation values of low importance, but also a group considering universalism values of utmost importance and opposing power values.

### Dutch politics and political orientation

For readers unfamiliar with the Dutch politics, we now describe developments in the Dutch political landscape over the last two decades. Important developments include both specific events and the emergence and disappearance of political parties.

As in other Western nations, Dutch politics have seen a number of important events during the first decade of the 21st century. A main development concerns the emergence of populist parties such as Liveable Netherlands (LN), TON, LPF and PVV. Recent evidence from other countries indicate that this trend is not unique to the Netherlands (e.g., the rise of Front National in France, and of the AfD in Germany). Specific events, such as the September 11 attacks in the United States in 2001, the assassination of the LPF leader Pim Fortuyn shortly before the general election in 2002 (May 6), and the murder of film director Theo van Gogh (November 2, 2004) have helped underscore the issues of the populist parties. Van Gogh, critical of aspects of Islam in his work, was murdered by a radical Dutch Muslim, sparking a number of retaliatory incidents.

For a long time there have been three main political parties in the Netherlands: CDA, PvdA and VVD (religious, libertarian/left and right respectively), which were linked to specific social groups (pillars) in society [21]. These three parties enjoyed over 80% of the votes in the 1989 elections. They still enjoyed over 60% of the votes in 2002. Since 2008, other parties have become larger, such as the left-wing Socialist Party (SP) (9.8% in 2010) and the populist PVV (15% in 2010). The growth of these latter parties came at the expense of the three long-established parties, making depillarization more visible. In the 2017 elections, five parties received only about 70% of the votes; the VVD, PVV, CDA, D66 and GL (Green Left) now belong to the larger parties. Another change during the last decade is that the number of small parties with a few seats in parliament is increasing because of the relative openness of the Dutch party system [20]. For instance, a party focused on animal welfare (PvdD) has five seats, and a party focused on the elderly (50PLUS) has four. Consequently, more parties are now represented in the Dutch parliament: the political landscape has become more diverse.

In the last years the larger old established parties, a few smaller parties with a specific program (e.g., PvdD focussing on animal rights) and the populist party (PVV) coexist. Also earlier models on electoral behavior, such as the heartland model, became less relevant [21] as people choose smaller parties that focus on topics that formerly belonged to the heartland of one of the three major parties (e.g, compulsory integration of ethnic minorities formerly belonging to the liberal heartland (VVD) is being taken by populist parties).

To describe the political arena in the Netherlands, three dimensions are identified [22]: left versus right, authoritarian versus libertarian and religious versus secular. In this context, left (right) indicates opposition to (support for) differences in social equality between people and support for (opposition to) a strong role for government in society. In particular, right is primarily opposed to the state having a strong influence on the economy: it considers private enterprise important and accepts inequality in society. Authoritarian versus libertarian primarily relates to dealing with people from other cultures and law enforcement. On the libertarian side, people are open to other cultures and non-conformist practices such as abortion or euthanasia, whereas authoritarians are more traditional, less open to foreign cultures and consider law enforcement to be important. Finally, the religion dimension concerns the role that the church should play in society on moral issues. These three dimensions are not independent, left coincides with libertarian and right with authoritarian. Note that these three dimensions are not the only option for describing the party system in the Netherlands—see for example [21], which suggests a dimension based in immigration.

In the Netherlands the religious dimension distinguishes the strictly religious SGP (Reformed Political Party), the CU (Christian Union) and to a lesser extent the CDA (Christian Democrats) from the other parties [22]. The libertarian versus authoritarian dimension distinguishes the D66 (Democrats 1966), GL (Green Left), PvdA and SP (both socialist parties) from the other parties. Finally, the left-right dimension distinguishes the VVD and the new populist parties, such as the PVV, from the other parties. In the next sections we will use this categorization of Dutch parties based on these larger political orientations: the religious (SGP, CU, CDA), libertarian left-wing (GL, D66, PvdD), left-wing (PvdA, SP), right-wing (VVD), and populist (LPF, LN, PVV, TON) categories. We will add non-voters as a separate category. Each of these (groups of) parties focuses on specific topics that might appeal to basic human values held by different segments of people in the electorate.

In the next section, we discuss three theory-based propositions regarding relations between value segments and political orientation.

### Propositions

In this study, the number and type of value segments that will emerge is purely data driven. Yet the literature points to some types of segments that will likely be uncovered. While the priority people give to specific human values are relatively stable over time [23], the political landscape in Europe, and in the Netherlands in particular, has changed due to the emergence and disappearance of political parties in recent years. Although we cannot discount that people’s values change, people’s values change at a much slower rate than the political landscape [24]. If indeed values are more stable than the political parties’ manifesto’s, changes in voting depend mostly on the political parties: For instance, when Green Left emphasizes universalism values more strongly than other parties, it will attract more votes considering this value important. When a new party starts emphasizing this value more strongly than Green Left, voters may switch to that new party.

The change in political landscape suggests that for some (segments of) voters their best-fitting political parties may have changed. As most people aim to achieve congruence between their values on the one hand and behaviour on the other [25], this implies that people may feel the need to change their vote.

Below we list our three main propositions:

1. We posit that people who highly value universalism and self-direction, and to a lesser extent benevolence, have a positive stance toward outgroups and people who are different from themselves, such as immigrants or refugees [26, 27]. They value equal opportunities and taking initiative. When individuals are driven by values such as universalism and self-direction, they will be less motivated to have control, status and be secure and safe. Consequently, values such as power and security will be considered less important. Such a value profile is congruent with programs of left-wing and libertarian political parties (e.g., on openness to ethnic minorities [21]) and was found for the Italian [5] and Israeli electorates [28]. We therefore posit that there is a segment considering benevolence, universalism, self-direction and stimulation of particular importance, and power and security values of low importance which predominantly votes for left or libertarian parties.
2. For people who consider the security values and the adjacent power values relatively more important, it is important to keep the status quo. They prefer a stable society in which traditions are respected and conflicts with existing social standards and expectations are minimized. When power, security, and tradition drive their attitudes and behaviours they are willing to accept that they need to trade-off these values with universalism and self-direction values. This means they are willing to accept less freedom and independence (de-emphasizing self-direction), to be able to live a familiar and safe environment (emphasizing security). For people holding such a value profile, there is a clear congruence with right-wing parties that emphasize maintenance of the status quo, acceptance of inequality in society and strict rules, which relate to power, security and tradition but oppose universalism and self-direction; previous studies on values and voting have shown this to be the case in other multi-party systems in Israel and Italy [27, 28]. Moreover, the right-wing parties also emphasize the negative side of immigration [21], which is compatible with security (and opposes universalism). We therefore posit that there is a segment relatively more emphasizing power and security values and opposing universalism values anticipated to vote for right-wing parties.
3. Religiousness is positively related to the values conformity and tradition [29]. Being strictly religious coincides with traditional (gender) roles, conformity to rules, and taking care of people in the in-group. Values such as tradition and conformity are held at the expense of opposite values such as hedonism and stimulation. In addition, the religious are expected to consider benevolence to be more important than universalism as benevolence values emphasizes the traditional in-group more. A resulting circular value profile, with tradition values important and stimulation unimportant, resonates with the values of the religious parties that consider the role of the church in society of key importance. Specifically, it is expected that there is a segment highlighting conformity, tradition, and security as highly important and considering the opposing values self-direction, stimulation, and hedonism as opposed to their values. In line with earlier findings in Israel on the link between tradition, conformity and security values and voting for religious parties [28], we posit finding a segment emphasizing those three values voting for religious parties.

In the aforementioned three segments, we assume people to have a clear distinct value profile on the ten values, with some values being important guiding principles and other values being opposed to these important values with the remaining values in between. However, we expect to find more segments. Some people may have less outspoken value preferences and may consider all values of about equal importance. Such a less distinct value profile may have a substantive or a methodological reason. Only a small part of the population may have outspoken value profiles in which clear trade-offs between values are made, which may be an adaptive tendency [10]. Moreover, it may be easier to function if one does not have very strong value preferences or rejections, as the number and severity of conflicts one encounters then may be less. A methodological reason may be that respondents may not differentiate between the value items because they find the items in the questionnaire too difficult or abstract, or are less motivated to fill in the questionnaire and just tick what is easiest, also called a satisficing response tendency [30]. Given this, we expect that there will be segments where people have value profiles that resemble the three aforementioned ones, but there may also be other segments; these latter segments may even reflect response styles rather than substantive content.

Finally, we expect to make some interesting findings about non-voters. In the analysis, we aim to find a value profile associated with deliberately not voting. There is very little research on the relation between values and not voting. An exception is the study in Italy by [18], which suggests that non-voters’ values may not resonate with the values existing political parties advocate. Their main finding is that Italian non-voters have a less outspoken value profile that falls in between that of left-wing and right-wing voters. In the Netherlands, universalism is advocated by libertarian left-wing; tradition by religious parties; and power and security by right-wing parties. Given the circular theoretical structure of values (see Fig 1), we expect that non-voters will consider other values than universalism, power, security and tradition as the most important guiding principles in their lives; we also expect them to have a less outspoken value profile with most values of about equal importance.

Summarizing, we expect to find a small number of segments of people differing in the importance they attach to the 10 human values. These segments are then linked to voting behavior. Our expectations are to uncover both three segments of people with clear value profiles, for which we formulated propositions, as well as other segments with potentially less pronounced profiles. There is no guarantee that all segments will be compatible with Schwartz’s theory.

### Response styles

Response styles have long been acknowledged as an issue in public opinion research [31–36]. When answering survey questions using rating scales, people use the scale in a different way. Some tend to use the positive side of the scale, whereas others prefer to spread the answers across all available response categories, and still others prefer using the extreme categories only. Such differences in scale use contaminate the findings as they do not reveal differences in substantive content. For instance, a person using the scale categories “1” and “2”, and another person using the categories “2” and “3” can have the same value profile; the only difference is in the average across all values. Such a difference needs to be corrected for. Furthermore, some response styles can be problematic such as a style where people hardly differentiate between items (e.g., use one scale category only). In values research correcting for response styles is a recommended procedure [15] and we use the LC-BML model [16] that enables us to separate response style from substantive content in segmentation studies. Next, we introduce this model.

### The LC-BML model

To determine value segments free from response styles, we use the latent-class bilinear multinomial logit model (LC-BML model) [16]. This model is a multivariate generalization of the standard multinomial (or baseline-category) logit model for multinomial responses [37], which in turn is the multicategory extension of logistic regression. The multinomial logit model describes the probability that a respondent answers a single item with a given rating. The LC-BML model combines such models for multiple items in order to describe the responses of a respondent on all items simultaneously. The input for the model are items measured on rating scales; in the model the rating categories are considered as nominal and thus categories such as “no answer” can be added.

Furthermore, the model allocates all respondents into two types of segments (or latent classes), namely values and response style segments. This is done by allowing the model parameters to vary between segments. The response style segments correct for the differences in response styles, while the value segments indicate differences in the importance attached to the respective values. Hence each person is assigned simultaneously to both a response style segment and a value segment by the LC-BML model.

We will now describe the LC-BML model in more detail requiring some notation. Let *Y*_*ij*_ be the random variable denoting the response of respondent *i*(*i* = 1, …, *N*) to item *j*. Index by *k*(*k* = 1, …, *K*) the common rating scale used for all *J* items. Missing responses are included by adding a dedicated ‘Missing’ category to the rating scale. Let there be *R* and *S* latent classes for the response style and value segments respectively. Supposing that *π*_*rs*_ is the prior probability that any respondent belongs to segments *r* and *s*, it follows that

$$\begin{array}{c} P\left(Y_{ij}=k\right)=\sum_{r=1}^{R}\sum_{s=1}^{S}\pi_{rs}P\left(Y_{ij}=k\;|\;r,s\right). \end{array}$$ (1)

In the LC-BML model, the segment-specific probabilities follow multinomial logit models such that

$$\begin{array}{c} P\left(Y_{ij}=k\;|\;r,s\right)=\frac{\exp(\eta_{ijk\;|\;r,s})}{\sum_{k=1}^{K}\exp\left(\eta_{ijk\;|\;r,s}\right)}, \end{array}$$ (2)

where *η*_*ijk*|*r*,*s*_ is a segment-specific linear predictor. The basic form of the linear predictor is

$$\begin{array}{c} \eta_{ijk\;|\;r,s}=\alpha_{k\;|\;r}+\sum_{l=1}^{L}\beta_{kl}^{{}^{\prime }}x_{il}+\gamma_{jk\;|\;s}. \end{array}$$ (3)

Here *α*_*k*|*r*_ is the attractiveness of rating *k* in response style segment *r*, ***x***_*il*_ is an indicator vector indicating which category of the (discretized) socio-demographic variable *l*(*l* = 1, …, *L*) person *i* belongs to, $\beta_{kl}^{{}^{\prime }}$ is the transpose of the vector of effects for this socio-demographic variable on category *k*, and *γ*_*jk*|*s*_ is the effect of rating *k* on item *j* in value segment *s*. Finally, a bilinear decomposition is applied to the parameters in Eq (3), a description of which is deferred to S1 File. This decomposition serves two purposes, namely to reduce the number of parameters to be estimated and to allows for all effects to be interpreted graphically in biplots. Note that the dimensionality *P* of this decomposition determines the dimensionality of these biplots.

For a given choice of the number of segments *R* and *S*, and dimensionality *P*, the LC-BML model is estimated by maximum likelihood via the Expectation-Maximization [38] algorithm. The likelihood contribution of person *i* is given by

$$\begin{array}{c} \sum_{r=1}^{R}\sum_{s=1}^{S}\pi_{rs}\prod_{j=1}^{J}\prod_{k=1}^{K}P{\left(Y_{ij}=k\;|\;r,s\right)}^{I(y_{ij}=k)}, \end{array}$$ (4)

with *y*_*ij*_ being the realized value of *Y*_*ij*_ and *I*(⋅) the indicator function. An important by-product of the estimation algorithm is the estimated posterior probabilities of each person belonging to each of the *R* × *S* latent classes, which can be calculated as

$$\begin{array}{c} \pi_{rs}\left(i\right)=\frac{\pi_{rs}\prod_{j=1}^{J}\prod_{k=1}^{K}P{\left(Y_{ij}=k\;|\;r,s\right)}^{I(y_{ij}=k)}}{\sum_{r=1}^{R}\sum_{s=1}^{S}\pi_{rs}\prod_{j=1}^{J}\prod_{k=1}^{K}P{\left(Y_{ij}=k\;|\;r,s\right)}^{I(y_{ij}=k)}}. \end{array}$$ (5)

The model gives a posterior measure of class membership for all individuals. These measures can for instance be aggregated over the respondents’ self-reported voting behaviour to establish how people within each segment voted. Information criteria, such as the Akaike Information Criterion (AIC) [39] or Schwarz’s Bayesian Information Criterion (BIC) [40] can be used to select the number of segments *R* and *S*, as well as the dimensionality *P*. An alternative is the graphical CHull procedure of [41]. We opt for a combination of the BIC and the CHull procedure. The BIC has been shown to work well in conjunction with the LC-BML model [16].

## Materials and methods

### Data

This study uses five rounds of the European Social Survey (ESS), namely those of 2002, 2004, 2006, 2008 and 2010. The ESS is a large scale high-quality cross-national survey conducted biennially in approximately 25 European countries, including the Netherlands [42]. The survey covers representative samples of individuals of 15 years or older who live in the respective nations, with in total 9741 respondents from the Netherlands.

For the current study, we use the 9511 Dutch respondents from the 5 ESS waves for which information on key variables were available. For details on data collection procedures, see www.europeansocialsurvey.org. The number of respondents per ESS wave were 2323, 1854, 1845, 1713 and 1776 respectively.

The ESS has a specific web page on confidentiality, which is available at http://www.europeansocialsurvey.org/about/privacy.html. It is ensured that the ESS data are fully anonymized before researchers can access it: “In accordance with data protection regulations in participating countries, only anonymous data are available to users. Before depositing data to NSD [Norwegian Centre for Research Data], each national team is responsible for checking their data with confidentiality in mind and to undertake the necessary measures to ensure anonymity of the data files and to foresee that anonymity is also maintained after merging of data files.”

### Measures

We use the 21 value items available in the ESS (Schwartz Portrait Values Questionnaire, abbreviated as PVQ21), which measures the 10 different values [15, 43]. Each of these values are measured by two items, except for universalism. Universalism has three items, because it covers both tolerance to people in the outgroup as well protection of nature. All items are gender-specific and operationalized by statements such as one of the items on self-direction: ‘Thinking up new ideas and being creative is important for her. She likes to do things in her own original way.’ See S1 Table for the complete list of all value statements. The answering scale for the PVQ-ESS is a 6-point Likert scale, ranging from 1 (‘Very much like me’) to 6 (‘Not like me at all’); the category 7 (“no answer”) was added when scores were missing. In total, 134 respondents were removed who supplied no information on the 21 value items.

We include demographic information on age, gender and education. Since discrete covariates are required for the LC-BML model, both years of education and age were discretized into three categories. For years of education, the categories were low (1 to 10 years), intermediate (11 to 15 years) and high (16+ years). These are comparable to the International Standard Classification of Education (ISCED) levels 1 and 2, 3 to 5, and 6 and above respectively—see the ISCED documentation for more details. Age was divided into the categories 15 to 34 years, 35 to 59 years and 60 to 96 years. Respondents with missing values for the above-mentioned socio-demographic variables were removed from the analysis (85 persons), together with respondents who used the same rating to answer all 21 value items (11 persons). After removing these respondents, 9511 observations were used in the LC-BML analysis. Note that although sampling weights are applicable in the ESS, it was not possible to apply them in the LC-BML analysis due to software limitations. We do however apply post-stratification weights in summary statistics, which corrects for the sampling design and unit non-response.

In the ESS, respondents were also asked to indicate whether they voted in the most recent elections for the Second Chamber of Parliament (the main legislative body of the Netherlands), and, if so, for which political party. For the 2002 survey, this concerned the Dutch elections of 6 May 2002, for 2004 the elections of 22 January 2003, for both 2006 and 2008 the elections of 22 November 2006 and for 2010 the elections of 9 June 2010. Data collection for the third ESS wave started in September 2006, before the election took place in November. Respondents interviewed before the election were asked who they intended to vote for. After the election they were asked who they actually voted for. Roughly half the sample were interviewed before the election. In the questionnaire, LN was still given as a voting option. However, LN disbanded before the 2006 elections, hence we do not consider LN when interpreting voting behavior after 2004. The official election results are given in S2 Table. Of the people who indicated that they voted, not all people disclosed the party they voted for. The disclosure rates for those who indicated that they voted were quite high at 97.9%, 96.6%, 95.1%, 96.4% and 96.0% respectively.

Note that voting is not mandatory in the Netherlands. In recent elections, roughly 75% to 80% of the electorate chose to vote for the Second Chamber of Parliament. The weighted proportion of eligible respondents in our sample who indicated that they voted is slightly higher at 86.4%, 82.4%, 83.3%, 86.1% and 84.4% for the 5 ESS waves respectively. For our analyses, we include an explicit category, in addition to the party voted for, for respondents who chose not to vote. In addition to the people who deliberately chose not to vote, approximately 7.1% of the sample was not eligible to vote, mostly on account of being younger than 18 at the time of the election. In the analyses on voting, i.e., the correspondence analysis and the multinomial logit model, we only include the group who deliberately chose not to vote.

### Analysis procedure

We start the analyses with estimating the value segments and response style segments with the LC-BML model. To enable the correction for response styles, all 21 value items are used as input for the analysis. We do not combine the items into the 10 value types. The latter is an advantage as we can still observe differences in topics considered important by the respondents; for instance, universalism includes not only equality of people but also care for nature and security includes both national security and security in the familiar environment. Next, we use correspondence analysis to determine and vizualize the relationships between value segments and voting over time. Finally, to assess whether value segments can be used to predict voting for specific groups of political parties, we perform multinomial logistic regression analysis.

When discussing voting results below, we will use weights to relate socio-demographics to voting over time. Since the results of the relevant elections are publicly available at the population level (see S2 Table), we use these to recalibrate the post-stratification weights from the ESS before analyzing the voting behavior. These recalibrated weights are constructed so that the proportion of votes for all competing parties as well as the proportion of nonvoters in the observed sample match the official election results as closely as possible. Iterative proportional fitting, also known as raking, was used to construct these weights separately for each wave of the ESS [44]. These weights are subsequently used whenever voting behavior is considered. This recalibration procedure also adjusts for the respondents not eligible to vote in the elections, as well as the small proportion who did vote but either chose not to disclose for which party they voted, or could not recall which party they supported. This is done by increasing the weights of the remaining respondents such that the total weight for the eligible voters equals that of the entire sample.

### Model selection

In order to select the most appropriate model, we fitted the LC-BML model for *R* = 1, 2, …, 20 response style segments, *S* = 1, 2, …, 12 value segments, and *P* = 1, 2 dimensions. In total 480 different models were considered; however 9 models did not converge in the allotted number of EM iterations (10 000) and were discarded. The EM algorithm is however only guaranteed to find a local optimum of the likelihood function. We therefore estimated each of these models for 20 different random starts to increase our chances of finding the global optimum. Only the start which resulted in the highest value of the likelihood function is retained.

We plotted the maximized log-likelihood values against the model degrees-of-freedom for the 471 models. The convex hull enclosing the cloud of points can then be determined. We reduced the model selection problem by considering only the models that lie on this hull, similar to the CHull prodecure [41]. These models can be considered to present a good trade-off between complexity and data fit. We selected the model with *R* = 20 response styles, *S* = 7 value segments and *P* = 2 dimensions from the remaining 23 models, since it is more parsimonious than the more complex models. Our chosen model obtained the fourth lowest BIC value, but used significantly fewer degrees-of-freedom than the other models in the top five, ranked according to BIC. We now proceed to describe and interpret the results from this model.

## Results

### Response styles

**Fig 2:**
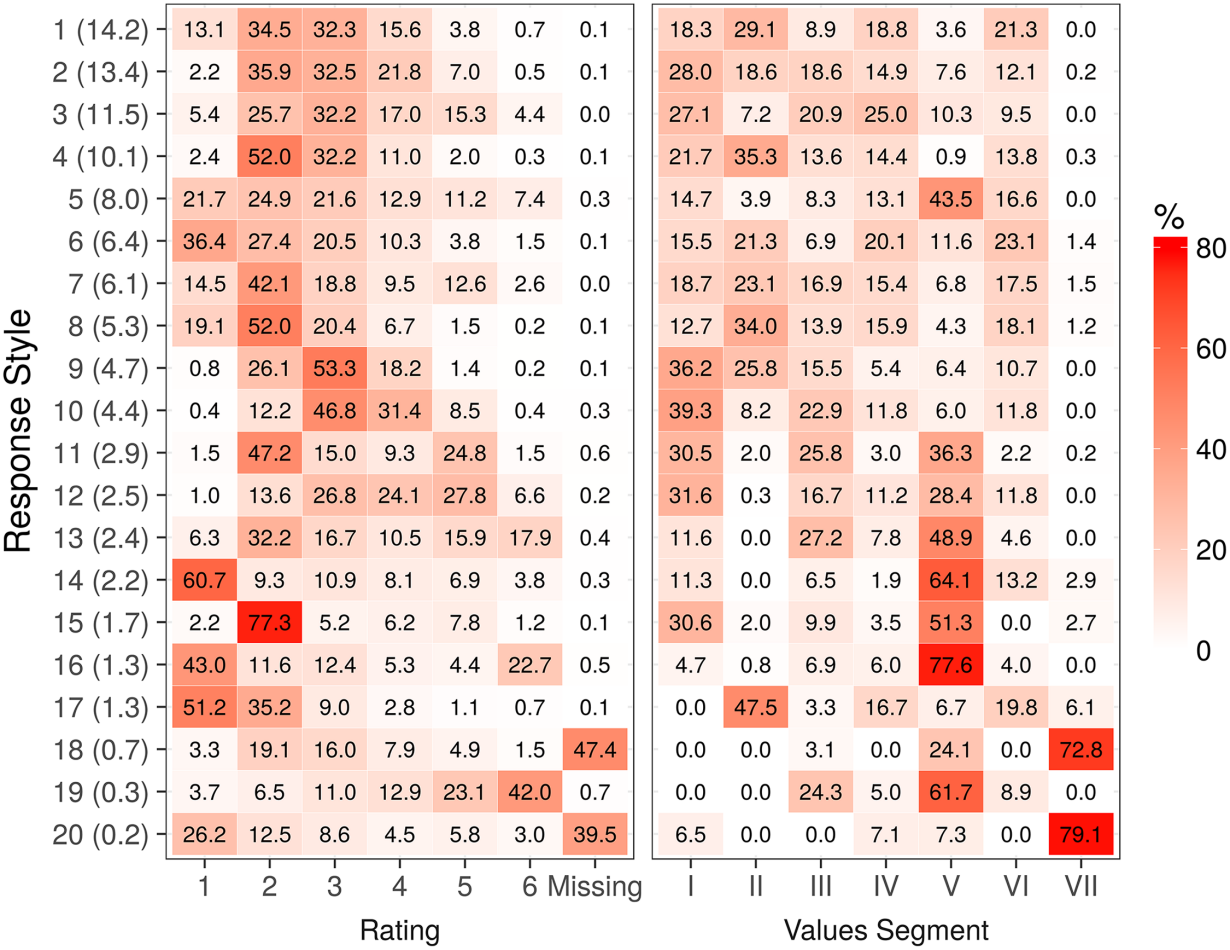
Response styles. The distribution of each response style segment across (1) all rating categories and (2) all value segments. The size of each response style segment, as a percentage, is given in parentheses after its number in the vertical axis labels. The table on the left shows the response categories used by the respondents to indicate their answers. The darker the cell, the more the respective rating category was chosen. For instance, in response style segment 4 (RS4 in the text), category “2” was chosen for 52% of the items and category “3” for 32.2% of the items. The table on the right shows combinations of response style segments and value segments. It can be seen that response style segments and value segments are not independent; for instance, response styles segments 18 and 20, with many missing answers, are more prevalent in value segment VII.

Our selected model has 2 dimensions, 20 response styles and 7 value segments. The response style segments indicate that respondents indeed differ in the way they use the rating scale for assessing the 21 value items. Fig 2 (left panel) gives the proportion of responses in each response style (RS) segment that was attributed to each of the ratings. Colours are used to highlight larger values. It is evident that a variety of response styles have been detected. The largest styles (RS1—RS3) concentrate on using ratings one through five, but very few sixes. RS4 focuses on using ratings two and three, while RS5 uses the breadth of the rating scale. RS9 and RS10 can be described as midpoint scoring, while RS16 comprises extreme scoring focusing on categories 1 and 6. RS18 and RS20 contain a disproportionate number of missing values. These results show that there is indeed substantial heterogeneity with respect to rating scale use.

Fig 2 also shows the association between the response style segments and value segments. The percentages in each row sums to one, showing which segments each response style is associated with. Some response style segments are often strongly associated with a single value segment. This is especially true for Segments V and VII. Specifically, Segment VII associates strongly with response styles RS18 and RS20 (missing values on many value items), and Segment V with RS13—RS16 and RS19. The latter include some well-known styles [13, for example], such as response range (RS13), acquiescence (RS14 and RS15), extreme responding (RS16) and disacquiescence (RS19). As Segment VII reflects a small segment of people (comprising 1.1% of the sample) who evidently skipped questions, we do not further describe this segment in the next sections. We will come back to the issues in Segment V when describing this segment in the next sections.

### Value segments

The bilinear decomposition in the LC-BML model represents the value segments in two dimensional perceptual maps or biplots. The first dimension distinguishes positive responses (1 = “very much like me”), from very negative responses (6 = “not like me at all”), with the other response categories arranged in between. The second dimension distinguishes responses in the middle of the rating scale indicating moderate importance (3 and 4) from the category “no answer”. From a substantive viewpoint, the first dimension is the most informative. We base the discussion below on this dimension only, using one-dimensional plots included in the main text, and defer the full two-dimensional plots to S1 Fig.

**Fig 3:**
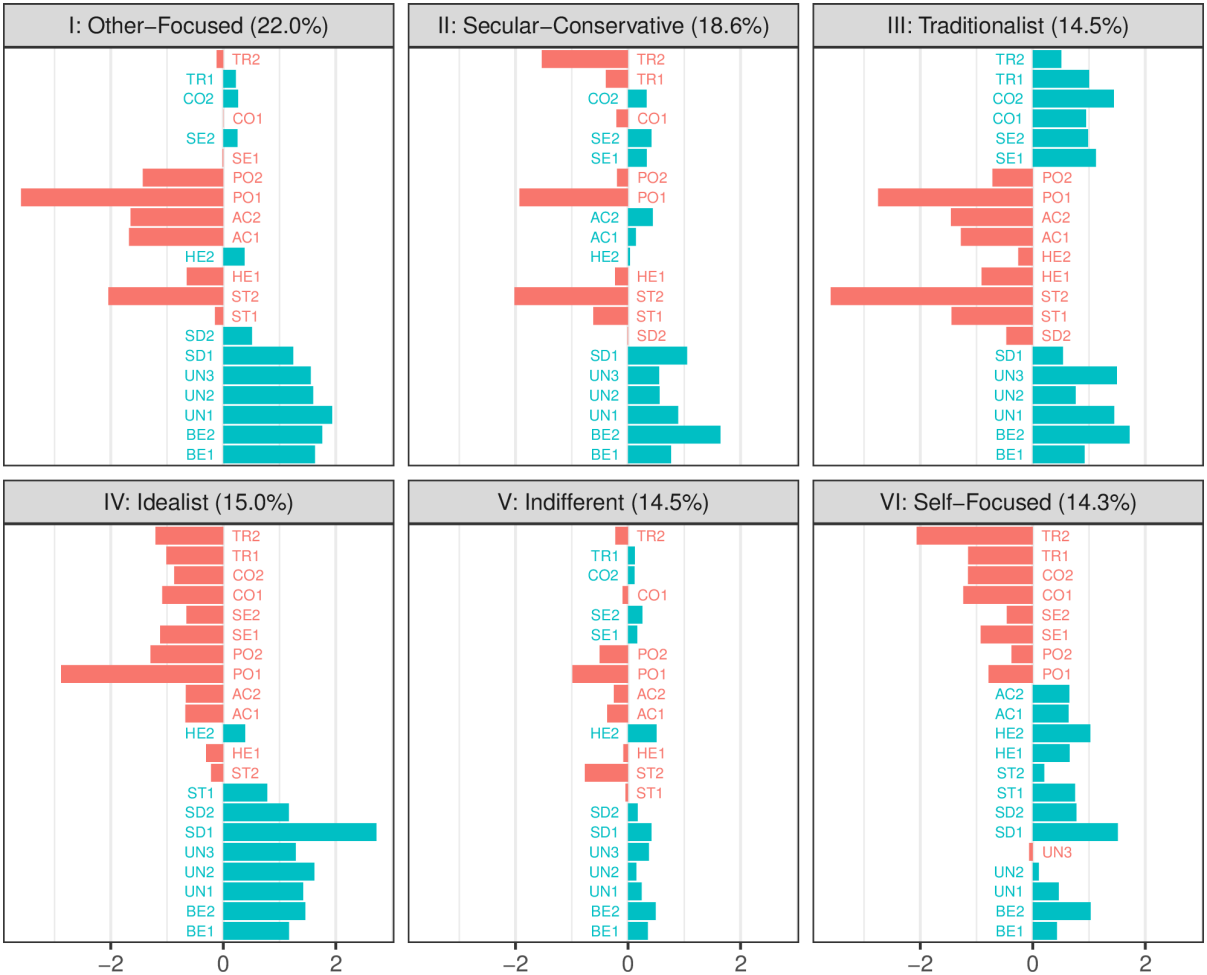
Barplots for the value segments I-VI. Barplots for the first six value segments. The value items are grouped by value (e.g., the two benevolence items, BE1 and BE2, are next to each other), and ordered according to Schwartz’ theory. For ease of interpretation, the bars are coloured green for values considered more important than average and red for those considered less important than average. The horizontal axis represents the projection onto the first dimension of the LC-BML results; the more different from zero, the more/less important the value item is.

The seven value segments, Segments I through VII, reveal different value priorities which can be seen in the barplots in Fig 3. For ease of interpretation, the bars are coloured green for values considered more important than average and red for those considered less important than average. The value items are grouped by value (e.g., the two benevolence items, BE1 and BE2, are next to each other), and ordered according to Schwartz’ theory. Next, we interpret each value segment, in descending order according to size.

Segment I (Other-Focused). Segment I in the left upper corner in Fig 3 is the largest group, comprising roughly 22.0% of the sample. From the figure, we see that this segment considers the values universalism, benevolence, and to a lesser extent self-direction as being important guiding principles in their lives. Values opposing these values such as power and achievement are scored as unimportant, which means being motivationally incompatible with their values. It is interesting to observe that in this segment stimulation and in particular the item on ‘living an exciting life’ (ST2), is considered to be completely opposed to their values. In the value profile of Segment I, the trade-off that may be experienced between the values benevolence (BE) and universalism (UN) versus power (PO) and achievement (AC) in Schwartz’ theory is clearly present.

Segment II (Secular Conservative). In comparison with Segment I, people in Segment II (18.6%) do have a less outspoken value profile that clearly follows the values theory. People in Segment II do not clearly oppose values as people in segment I do. However, in comparison to the other segments, they consider the values self-direction (independence), security and achievement as relatively important, whereas tradition (and humility in particular) is considered opposed to their values.

Segment III (Traditionalist). Compared to the other segments, people in Segment III (14.5%) consider the conservation values (security, conformity and tradition) far more important as guiding principles in their lives than the other segments do. They also oppose the openness values, and stimulation and hedonism in particular. The value priorities of people in this segment follow the circumplex structure. They make a trade-off between conservation values and openness values; for instance, to be able to live properly and follow the customs handed down by religion or family (CO2 and TR1) they are willing to give up an exciting life (ST2) and looking for new things to do in life (ST1).

Segment IV (Idealist). People in Segment IV (15.0%) place relatively great emphasis on self-direction (SD1, independence), universalism (and UN2, understanding others different from them in particular). Tradition (TR) and conformity (CO) are opposed to these values. Values representing making independent decisions are the main motivation of people in this segment; power and being rich is not important. A clear trade-off is made between these sets of values, as can be seen in Fig 3. Being independent, open to others and being loyal to friends are key values and to attain this, people in segment IV are willing to give up living in a secure environment and being rich.

Segment V (Indifferent). This segment, comprising 14.5% of the sample, hardly differentiates between the values. They consider all values of about equal importance. As most other segments, they consider benevolence and universalism values more and power and achievement values less important, but they do not have clear outspoken value preferences nor do they make clear trade-offs between values.

Segment VI (Self-Focused). People in Segment VI (14.3%) consider the values self-direction, stimulation, hedonism and achievement as important motivational goals in their lives. Of the two benevolence values, only being loyal to friends is relatively more important. It should be noted that the values considered important are all focused on own pleasure and success. This can also be seen in the importance, relative to other segments, attached to being rich and being respected by others. Compatible with this, universalism is considered a far less important motivational goal. Furthermore this is the only segment in which taking care of nature and the environment (UN3) is considered unimportant.

Information on socio-demographics and the evolution of value segment sizes over time can be found in S2 File. Next, we consider how the relations between value segments and voting developed over time.

### Value segments and voting over time

**Fig 4:**
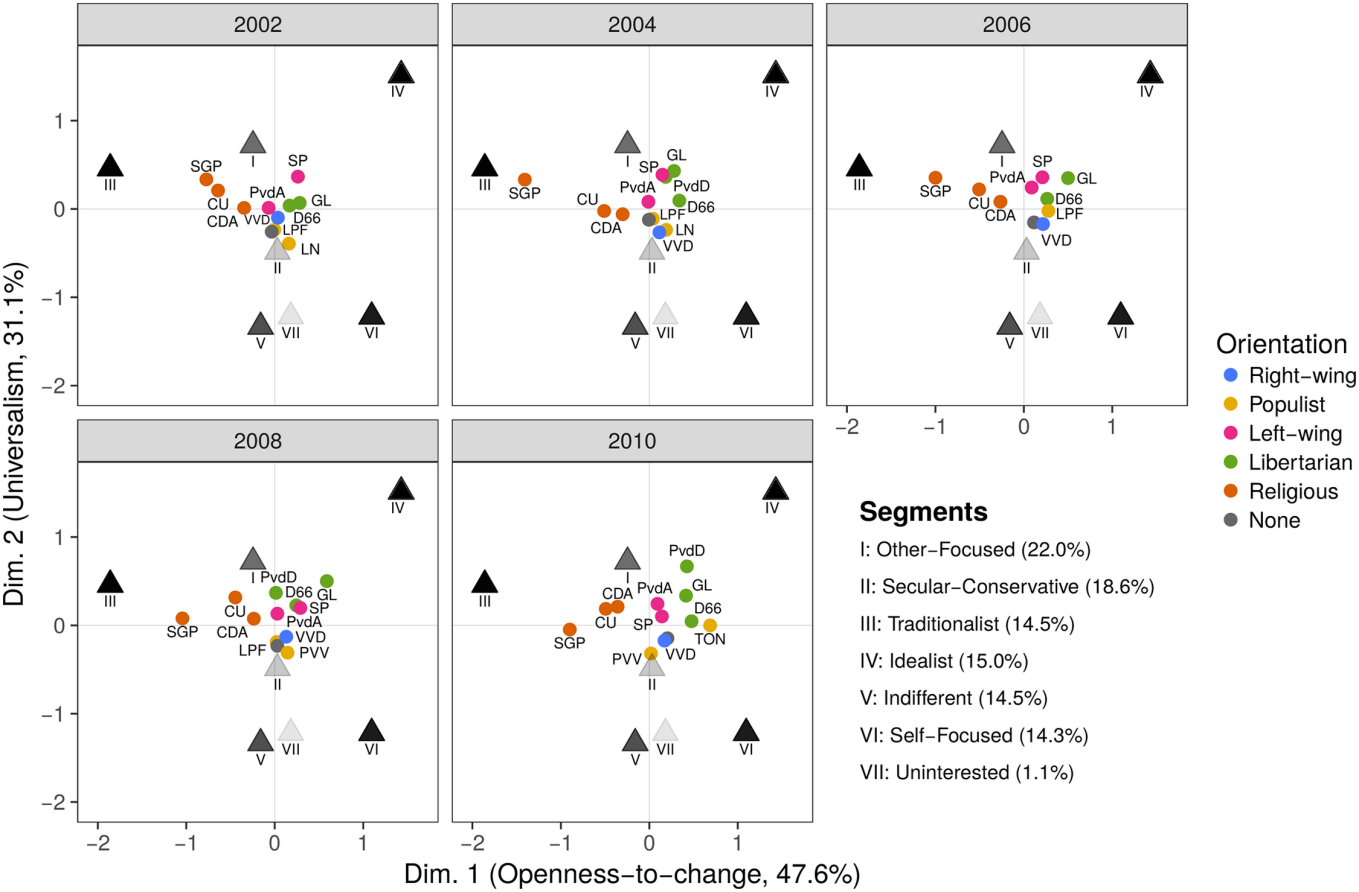
Correspondence analysis for the segments and reported votes. Correspondence analysis showing the graphical representation of the cross tabulation for the segments and reported votes over the years, using the adjusted poststratification weights applied to the posterior probabilities of Eq (5). The symbols for the segments are faded according to the explained inertia so that darker points fit better. No shading was done for the political parties. For clarity, each panel shows a different year, but the results are from the same single analysis. S2 Fig shows all years in a single plot. The first dimension can be labeled ‘openness-to-change’ and the second dimension ‘universalism’. The closer a party is to a segment, the more likely it is that this segment will vote for the party. For instance, dimension one distinguishes the traditional segment (III) from the other segments; Segment III is more likely to vote for SGP or CU and less likely to vote for GL. The plots also show that the positions of the political parties relative to the segments changes over time.

To gain insight into the relationship between value segments and voting over time, we performed a correspondence analysis (CA) [45, 46]. CA can be used to analyse and vizualize the cross-tabulation of the value segments together with the political parties for which they voted. This makes it easier to interpret our results. For this analysis, a cross-tabulation of the individual posterior probabilities in Eq (5) was made with the value segments in the columns and the political parties, split into the five waves of the ESS, in the rows. The CA was used to assess the links between the rows and columns in this table simultaneously (see Fig 4). The results in the table can be shown effectively in two dimensions (with a fit of 78.6% in two dimensions).

Fig 4 shows the column-principal map of the CA—see [46] for details—where each of the political parties are labelled, and each year is plotted in a separate panel. We use colours to categorize the parties according to their background: green for religious, red for socialist, blue for right-wing political parties, black for the populist parties, and yellow for the libertarian parties. The black triangles represent the seven value segments. The darker the points, the better the explained inertia (fit) of the points—see [46] for more information on inertia. Across the five waves, three value segments are quite distinct, namely the Segments III, IV, and VI. Segment I is located in between the Segments III and IV and lies more towards the centre of the graph. Segments V and VII lie close together; however, Segment VII fits less well into the CA solution, suggesting possible measurement issues in this segment. Last, Segment II also fit less well into the display and should therefore not be strongly interpreted.

The first dimension divides Segment III from Segments IV and VI. In value terms this is a distinction between having an emphasis on the three values conformity, tradition, and security or de-emphasizing these values. The second dimension is a distinction between Segment IV, and to a lesser extent Segment III, and Segment VI. This is a distinction between an emphasis on universalism values versus achievement values. The first dimension distinguishes the religious parties (SGP, CU and CDA) from the non-religious parties (e.g., GL, D66 and VVD). The second dimension distinguishes the libertarian parties (GL and PvdD) from the right-wing party (VVD) and the populist parties.

The religious parties lie in the upper left quadrant, strongly associated with Segment III. They are ordered from the most traditional SGP to the more moderate CDA, and are clearly distinguished from the libertarian and authoritarian parties towards the right of the graph. The libertarian and left parties, namely the PvdD, D66, GL and PvdA, are located alongside each other to the top right quadrant of the figure. The more strict libertarian left-wing parties, the GL and PvdD, are located in the upper right corner, whereas the more centrist PvdA is located towards the origin. The populist and right wing parties form a tight cluster slightly below the centre. These parties (LPF, PVV, VVD and LN) have tight historical connections, as for instance the populist LPF was formed by a former member of LN, and the PVV leader similarly was a former member of parliament for the right-wing VVD.

In a volatile political landscape, the party voted for may change over time and and we postulate that segments vote for the party most congruent with their values. Contrary to basic human values, political values are less stable over time; in particular, basic human values underlie specific political values [3]. In Fig 4, the religious parties hardly change position over time: they remain closest to Segment III over the years. The left-wing parties (PvdA and SP) have moved towards the libertarian parties and closer to each other. The right-wing party remained at about the same position over time; however, it is also this position that some populist parties have taken. The only populist party that took a position in between segments IV and VI appeared (and disappeared) in 2010. Finally, the people who deliberately did not vote are located close to the populist parties; this position hardly changed over time.

In the final results section, we show that our value segments are predictive of voting.

### Predicting voting with value segments

To validate the value segments obtained and discussed above, we performed a multinomial logistic regression analysis to predict voting using value segment membership probabilities. In this analysis, we use only the 8808 individuals who were eligible to vote, and who either revealed the party they voted for using the categories listed on the ESS questionnaire (all parties except ‘other’, blank, refusal and ‘don’t know’) or indicated that they deliberately did not vote. The dependent variable has six categories: five categories for political orientation (left-wing, libertarian, populist, religious, or right-wing), and the sixth category is reserved for non-voters. Also, combining parties ensures that each category has enough observations to be modelled. The independent variables used are socio-demographics, the ESS wave number (with wave 2002 as a reference), and the value segment membership probabilities. We estimate nested models to provide insight into the additional predictive value of each step.

Table 1 compares multinomial logit models using likelihood ratio tests. The models are listed in order of increasing complexity. All the effects included are main effects. The results show that the value segments add to model fit over and above demographics and ESS waves.

**Table 1:** Analysis of variance for multinomial logit models estimating the parties voted for.

	Model	Resid. df	Resid. Dev	Test	Df	LR stat.	p–value
1	Intercept only	44035	31045.8				
2	Demographics	44010	30063.4	1 vs 2	25	982.41	<0.001
3	Demographics, ESS wave	43990	29311.9	2 vs 3	20	751.47	<0.001
4	Demographics, ESS wave, Segments	43960	28531.7	3 vs 4	30	780.18	<0.001

Analysis of variance table for five multinomial logit models estimating the parties voted for by ESS respondents. Recalibrated post-stratification weights were used for all models, with only the 8841 persons who disclosed their vote being used. Model 4 uses the value segments membership probabilities derived from the LC-BML model fit. The abbreviations used are: df = degrees-of-freedom; Resid. Dev = residual deviance; LR stat = likelihood-ratio statistic.

The results are shown in Table 2. Differences in voting for political parties can be explained by both socio-demographics and value segment membership. Voters are higher educated and older than non-voters (coefficients for education and age are negative and significant); only voters for the populist parties are more similar to the non-voters in age. Voters for the right wing party are more often male (.21, *p* < .01) and less often belong to the low and middle education categories (-1.93 and -.84, both *p* < .001). The coefficients for the waves show whether a political orientation gained or lost votes over the years. These results show that in the 2010 election the religious parties lost votes to non-voters (reference category). Furthermore, after 2002 populist parties lost votes, but this trend is turning in 2010.

**Table 2:** Estimated coefficients for the multinomial logit model fitted to voting behaviour.

	Left-wing			Libertarian			Populist			Religious			Right-wing		
	$\hat{\beta }$	SE	p	$\hat{\beta }$	SE	p	$\hat{\beta }$	SE	p	$\hat{\beta }$	SE	p	$\hat{\beta }$	SE	p
Intercept	0.86	0.32	0.008	-0.20	0.64	0.757	0.13	0.41	0.758	1.22	0.34	<0.001	0.66	0.41	0.111
Gender (Male)	-0.00	0.07	0.975	-0.17	0.09	0.054	0.03	0.09	0.781	0.09	0.07	0.163	0.21	0.08	0.006
Education (1–10)	-1.14	0.10	<0.001	-2.12	0.15	<0.001	-0.59	0.14	<0.001	-1.29	0.10	<0.001	-1.93	0.12	<0.001
Education (11–15)	-0.82	0.09	<0.001	-1.32	0.11	<0.001	-0.35	0.12	0.004	-0.72	0.09	<0.001	-0.84	0.09	<0.001
Age (15–34)	-1.26	0.10	<0.001	-1.22	0.15	<0.001	-0.62	0.15	<0.001	-1.50	0.10	<0.001	-1.93	0.12	<0.001
Age (35–59)	-0.42	0.09	<0.001	-0.39	0.13	0.002	-0.06	0.13	0.617	-0.77	0.09	<0.001	-0.80	0.10	<0.001
Wave (2004)	0.55	0.10	<0.001	-0.09	0.13	0.501	-1.05	0.14	<0.001	0.10	0.10	0.299	0.25	0.12	0.029
Wave (2006)	0.62	0.10	<0.001	-0.72	0.15	<0.001	-4.41	0.60	<0.001	0.10	0.10	0.326	0.04	0.12	0.743
Wave (2008)	0.58	0.10	<0.001	-0.44	0.14	0.002	-1.05	0.14	<0.001	0.04	0.10	0.674	-0.08	0.12	0.501
Wave (2010)	-0.00	0.10	0.974	-0.20	0.12	0.102	-0.39	0.11	<0.001	-0.84	0.10	<0.001	-0.09	0.11	0.407
I: Other-Focused	0.77	0.31	0.014	1.70	0.63	0.007	0.17	0.40	0.664	0.97	0.33	0.004	0.57	0.41	0.161
II: Secular-Conservative	0.36	0.32	0.257	1.31	0.64	0.040	0.42	0.40	0.289	0.70	0.34	0.038	1.21	0.41	0.003
III: Traditionalist	0.17	0.32	0.591	0.73	0.65	0.261	-0.23	0.41	0.582	1.26	0.34	<0.001	0.33	0.41	0.428
IV: Idealist	0.93	0.32	0.003	2.19	0.63	0.001	-0.29	0.42	0.479	-0.23	0.35	0.512	0.38	0.41	0.354
V: Indifferent	-0.53	0.32	0.098	0.02	0.65	0.978	0.19	0.40	0.630	-0.17	0.34	0.612	-0.01	0.41	0.985
VI: Self-Focused	-0.12	0.32	0.716	1.37	0.64	0.032	0.26	0.40	0.513	-0.21	0.35	0.547	1.16	0.41	0.004

Results for the multinomial logit model fitted to voting behaviour. The model summarized here is model 4 in Table 1. The reference category for the response variables is the non-voters. The remaining five categories are shown in different sets of columns. The abbreviations used are: $\hat{\beta }$ = estimated regression coefficient; SE = standard error of the coefficient; p = p-value for testing whether the coefficient differs significantly from zero or not. The reference categories for the categorical variables are not shown in the table. These are: Gender (Female), Education (16+ years), Age (60–96), Wave (2002) and Segment VII.

The seven value segments help predict which group of parties is voted for. People belonging to segment I vote either for the left-wing parties (.77, *p* < .01), for libertarian (1.70, *p* < .01) or for the religious parties (.97, *p* < .001), whereas people in segment II tend to vote right-wing (1.21, *p* < .001) or libertarian (1.31, *p* < .05). Segment III, with strong traditional values, prefers the religious parties over the other parties (1.26, *p* < .001). Segment IV votes for either left wing (.93, *p* < .001) or libertarian parties (2.19, *p* < .001). Segment V is interesting as people in this segment seem to be against voting; we can see that the coefficients do not differ from the non-voters. Lastly segment VI votes for the right wing party (1.16, *p* < .001) or for one of the libertarian parties (1.37, *p* < .05). The libertarian parties compete for voters from four segments (I, II, IV and VI); the religious parties obtain voters mainly from one segment (III).

We posited that we would find a segment emphasizing universalism, self-direction and stimulation values and opposing power and security values voting for libertarian parties; segment IV has a strong preference for libertarian parties, confirming the first proposition. This result is in line with the earlier findings in Italy [5]. We further posited that there would be a segment emphasizing power and security values, and opposing universalism values, voting for right-wing parties. We did not find such a segment; therefore could not confirm this proposition. We also find that there is a segment (III, Traditionalist) emphasizing the three values security, conformity and tradition, opposing the values self-direction, stimulation and hedonism, and which considers benevolence a stronger guiding principle than universalism, voting for religious parties. In line with earlier research in Israel [28], we thus confirm our third proposition.

## Discussion and conclusion

There is a clear link between the seven value segments based on Schwartz’ values and people’s voting behaviour for political parties. Our approach using the LC-BML model, followed by a multinonial regression and a correspondence analysis, clearly reveals distinct value segments that are attracted to different political parties. The religious, left-wing/libertarian and right-wing parties each appeal to people with different distinct value profiles, each following Schwartz’ theoretical framework [3]. People with less outspoken value profiles feel attracted to the populist parties or do not vote.

This study distinguishes itself from previous studies on human values and voting in multiple ways. First, we focus on segments instead of considering the whole population as one group. Specifically, we relate value segments to voting for specific political parties. With our approach we are not only able to confirm established relationships between single values and political orientation, such as a correlation between the value universalism and a left-wing orientation [5], but also to determine which combinations of values are related to voting behaviour. In this we confirm the existence of a values structure showing the oppositions and compatibities between values of an individual [10] and are able to relate this to specific political orientations. Second, we correct for response styles, which is a neglected issue in research on human values and voting. Third, our study is the first that shows the relationship between values and voting behaviour over a period longer than a decade; the earlier longitudinal study [27] covered one month. Fourth, we treat all 21 value items separately, instead of reducing the items to the 10 values they set out to measure [5, 47], or to their respective higher-order value domains, such as self-transcendence [28]. We are able to show that value segments not only differ with respect to the importance they attach to (higher-order) values, but also with respect to specific items. For example, in most segments the two items measuring power are far apart. Also segments distinguish themselves on universalism focused on nature, separating segment VI (Self-Focused) from the other segments; the latter result would have been hidden when all universalism items would have been combined in one score for universalism.

The LC-BML model identified 20 different response style segments, providing further empirical evidence of the prevalence of response styles in rating scale data. Importantly, our results show that the value segments and response style segments are not independent. Two more problematic value segments, namely Segments V (Indifferent) and VII (Uninterested) in particular, are closely associated with specific response styles. Together, these comprise 15.7% of the sample. Hence the answers of a significant number of respondents are mainly driven by specific response styles. In the other segments we might consider the way in which people use the rating scale a communication style. With a communication style, no adjustment, or simple adjustments, may suffice [48]. Our study indicates that taking into account response styles in value measurement is important, since ignoring response styles can lead to segments that differ only with respect to rating scale use, and not in value preference. Our study also suggests that the response styles present in empirical data are not limited to one specific style such as extreme responding: people use many different response styles that all might invalidate our findings [49, 50].

The relations between the value segments and voting behaviour are quite stable over time. This is in line with the literature that also shows that when change occurs the hierarchy of importance of the values the relative order of importance of the values stays about similar [51]. As evidenced in the correspondence analysis (Fig 4), specific segments tend to vote for specific (sets of) political parties. For example, segment III tends to vote for the religious parties (SGP, CU and CDA) relatively frequently, whereas segment VI votes more frequently for the right wing party, VVD, or a libertarian party and people in segment IV choose one of the left or libertarian parties.

A remarkable segment is Segment V. This segment either tends not to vote, or, when voting, likely NOT votes for one of the established parties. They might vote for a populist party, but that is not shown in our results. Looking at the literature, a reason for their (non)-voting might be value incongruence with the existing parties, as suggested for Italian voters [18]. However, the value profile of segment V is not very clear; they consider all values of about equal importance. Value incongruence with specific parties then might be less obvious as we found that they are less likely to vote for all political orientations. Another possibility is that people in Segment V have differentiated less between items in the survey, which might be due to less education or lack of motivation to answer the items [52]. Still, Segment V is a stable segment in our correspondence analysis, indicating that such a segment is valid and stable and will likely not vote, or when they vote may choose one of the newly emerging (populist) parties.

Our results also pose interesting questions for further research. For example, we have seen that Segment III has shrunk between 2002 and 2010, while segment IV has grown over the same period. In that period, the Netherlands has become more prosperous, as evidenced by the growth in the GDP per capita. Increased prosperity leads people to consider postmodernist values such as independence to be more important [53], making a segment considering universalism values important larger. Further research might focus on value change and the effects that might have on the size of our respective value segments and on voting behaviour. The LC-BML model is able to detect groups of persons who did not provide much substantive information in their responses, as evidenced by Segments V and VII. For these segments, it is difficult to gauge their true opinions from the data. This is a significant finding since it shows that also in well-designed surveys advanced analysis methods can only extract limited information for a substantial number of respondents. Further research might focus on determining why such persons answer in the way they do, and how survey design elements can be used to counteract this type of non-informative responding. Fortunately, these persons tend to answer using a small set of typical response styles, enabling survey researchers to detect them.

Finally, it should be noted that the ESS is not an election survey and therefore has some potential limitations. It is not longitudinal, as each wave has a different sample of respondents. Another limitation is that the length of time since the last election differs from wave to wave. For example, the 2002 wave refers to the 2002 election, whilst both the 2006 and the 2008 waves refer to the election of 2006. A concern might be that voters do not properly recall their voting behaviour after a prolonged period of time. Regarding recall of votes, it is known from research on values and voting that even 27 months after an election the relationship between values and parties voted for is largely similar to the relationship just after the election [5].

## Supporting information

S1 File Bilinear decompositions in the LC-BML model. A mathematical description of the bilinear decompositions used in the LC-BML model.
(PDF)

S1 Table Items used in Schwartz’ PVQ values scale. A description of the items used in Schwartz’s PVQ values scale, together with the average ratings.
(PDF)

S2 Table The official Dutch national election results. An overview of the Dutch election results for the period 2002—2017.
(PDF)

S1 Fig The biplots produced by the LC-BML model. The full plots from which Fig 3 is derived.
(PDF)

S2 File Value segments, socio-demographics and stability over time. A discussion of the relationship between socio-demographics and the value segments, and time trends.
(PDF)

S2 Fig Correspondence analysis plot of value segments and voting behaviour. Similar to Fig 4, but with all the ESS waves displayed in a single plot.
(PDF)
