# Supplementary material for: Competing for the same value segments? Insight into the volatile Dutch political landscape
Source: PLoS One. 2018 Jan 11;13(1):e0190598. doi: 10.1371/journal.pone.0190598 (PMC5764305; doi:10.1371/journal.pone.0190598)
Supplement: S1 File — A mathematical description of the bilinear decompositions used in the LC-BML model. (PDF) [file pone.0190598.s001.pdf]

Bilinear Decompositions in the LC-BML Model

We now describe the bilinear decomposition of the parameters used in the LC-BML model. In order to reduce the number of parameters in Eq. (3) from the main text, which reads

$$\eta_{ijk\,|\,r,s} = \alpha_{k\,|\,r} + \sum_{l=1}^{L} \boldsymbol{\beta}^{'}_{kl}\boldsymbol{x}_{il} + \gamma_{jk\,|\,s}, \tag{1}$$

[1] introduce bilinear decompositions which also make it possible to display all effects in P-dimensional graphs. Let $\mathbf{B}_l$ be the matrix with $\boldsymbol{\beta}_{1l}, \ldots, \boldsymbol{\beta}_{Kl}$ as rows, and gather the $\gamma_{jk\,|\,s}$ in the $J \times K$ matrix $\boldsymbol{\Gamma}_s$. The bilinear restrictions are imposed by requiring that

$$\mathbf{B}_l = \mathbf{F}\mathbf{G}^{'}_l \text{ and } \boldsymbol{\Gamma}^{'}_s = \mathbf{F}\mathbf{H}^{'}_s, \tag{2}$$

where $\mathbf{F}$, $\mathbf{G}_l$ and $\mathbf{H}_s$ has P columns. Typically, the dimensionality of the graphical representations is chosen to be $P = 1$, 2 or 3 so that it can be displayed easily. The matrix $\mathbf{F}$ contains the coordinates of the K rating categories, $\mathbf{G}_l$ the coordinates for the categories of socio-demographic variable l, and $\mathbf{H}_s$ the coordinates of the J items in value segment s in P-dimensional space. Under these bilinear restrictions, Eq. (1) becomes

$$\eta_{ijk\,|\,r,s} = \alpha_{k\,|\,r} + \sum_{l=1}^{L}\sum_{p=1}^{P} f_{kp}\boldsymbol{g}^{'}_{lp}\boldsymbol{x}_{il} + \sum_{p=1}^{P} f_{kp}h_{jp\,|\,s}, \tag{3}$$

with f_{kp} and $h_{tp\,|\,s}$ being the elements of $\mathbf{F}$ and $\mathbf{H}_s$ respectively, and $\boldsymbol{g}_{lp}$ the pth column of $\mathbf{G}_l$. Besides these bilinear restrictions, several identifiability contraints must be imposed on the parameters in Eq. (3) – details are given in [1].

References

[1] Van Rosmalen, Joost, Hester Van Herk, and Patrick JF Groenen, 2010. "Identifying Response Styles: A Latent-Class Bilinear Multinomial Logit Model." *Journal of Marketing Research* 47:157–172.
