# Supplementary material for: Competing for the same value segments? Insight into the volatile Dutch political landscape
Source: PLoS One. 2018 Jan 11;13(1):e0190598. doi: 10.1371/journal.pone.0190598 (PMC5764305; doi:10.1371/journal.pone.0190598)
Supplement: S1 Table — A description of the items used in Schwartz’s PVQ values scale, together with the average ratings. (PDF) [file pone.0190598.s002.pdf]

Value	Code	Item	Description	Average
Benevolence	BE1	It's very important to him to help the people around him. He wants to care for their well-being.	Help others	2.27
	BE2	It is important to her to be loyal to her friends. She wants to devote herself to people close to her.	Loyalty	2.16
Universalism	UN1	He thinks it is important that every person in the world should be treated equally. He believes everyone should have equal opportunities in life.	Equality	2.11
	UN2	It is important to him to listen to people who are different from him. Even when he disagrees with them, he still wants to understand them.	Understand others	2.43
	UN3	She strongly believes that people should care for nature. Looking after the environment is important to her.	Care for nature	2.31
Self-Direction	SD1	It is important to her to make her own decisions about what she does. She likes to be free and not depend on others.	Independent	2.11
	SD2	Thinking up new ideas and being creative is important to her. She likes to do things in her own original way.	Creative	2.46
Stimulation	ST1	He likes surprises and is always looking for new things to do. He thinks it is important to do lots of different things in life.	Look for new things	2.85
	ST2	He looks for adventures and likes to take risks. He wants to have an exciting life.	Excitement	3.72
Hedonism	HE1	Having a good time is important to her. She likes to spoil herself.	Have a good time	3.04
	HE2	He seeks every chance he can to have fun. It is important to him to do things that give him pleasure.	Have fun	2.52
Achievement	AC1	It's important to her to show her abilities. She wants people to admire what she does.	Be admired	3.27
	AC2	Being very successful is important to him. He hopes people will recognise his achievements.	Be successful	3.26
Power	PO1	It is important to her to be rich. She wants to have a lot of money and expensive things.	Be rich	4.18
	PO2	It is important to her to get respect from others. She wants people to do what she says.	Get respect	3.40
Security	SE1	It is important to him to live in secure surroundings. He avoids anything that might endanger his safety.	Security	2.80
	SE2	It is important to her that the government ensures her safety against all threats. She wants the state to be strong so it can defend its citizens.	Strong government	2.73
Conformity	CO1	He believes that people should do what they're told. He thinks people should follow rules at all times, even when no-one is watching.	Follow rules	2.95
	CO2	It is important to her always to behave properly. She wants to avoid doing anything people would say is wrong.	Behave properly	2.85
Tradition	TR1	Tradition is important to her. She tries to follow the customs handed down by her religion or her family.	Tradition	2.86
	TR2	It is important to him to be humble and modest. He tries not to draw attention to himself.	Modesty	3.29

Table S2.1: The 21 value items from the ESS and their weighted average ratings for the Dutch sample. The unadjusted post-stratification weights supplied in the ESS were used to calculate the weighted averages. A six point scale was employed, with one being the highest possible score, and six being the lowest possible score.
