# Supplementary material for: Competing for the same value segments? Insight into the volatile Dutch political landscape
Source: PLoS One. 2018 Jan 11;13(1):e0190598. doi: 10.1371/journal.pone.0190598 (PMC5764305; doi:10.1371/journal.pone.0190598)
Supplement: S2 Table — An overview of the Dutch election results for the period 2002—2017. (PDF) [file pone.0190598.s003.pdf]

	Election					
**Party**	**2002**	**2003**	**2006**	**2010**	**2012**	**2017**
VVD	15.4	17.9	14.7	20.5	26.6	21.3
PvdA	15.1	27.3	21.2	19.6	24.8	5.7
PVV	–	–	5.9	15.4	10.1	13.1
CDA	27.9	28.6	26.5	13.6	8.5	12.4
SP	5.9	6.3	16.6	9.8	9.7	9.1
D66	5.1	4.1	2.0	6.9	8.0	12.2
GL	7.0	5.1	4.6	6.7	2.3	9.1
CU	2.5	2.1	4.0	3.2	3.1	3.4
SGP	1.7	1.6	1.6	1.7	2.1	2.1
PvdD	–	0.5	1.8	1.3	1.9	3.2
50PLUS	–	–	–	–	1.9	3.1
TON	–	–	–	0.6	–	–
LPF	17.0	5.7	0.2	–	–	–
LN	1.6	0.4	–	–	–	–
DENK	–	–	–	–	–	2.1
FvD	–	–	–	–	–	1.8
Other	0.7	0.4	1.0	0.5	0.9	1.5
Valid votes	9 501 152	9 654 475	9 838 683	9 416 001	9 424 235	10 516 041
Blank/Invalid	14 074	12 127	16 315	26 976	37 988	47 415
Total votes	9 515 226	9 666 602	9 854 998	9 442 977	9 462 223	10 563 456
Electorate	12 035 935	12 076 711	12 264 503	12 524 152	12 689 810	12 893 466
Turnout	79.1%	80%	80.4%	75.4%	74.6%	81.9%

Table S3.1: The percentage of votes won by the different political parties in the six Dutch elections, as well as the total number of votes cast and the size of the electorate. (Source: www.verkiezingsuitslagen.nl)
