# Supplementary material for: Competing for the same value segments? Insight into the volatile Dutch political landscape
Source: PLoS One. 2018 Jan 11;13(1):e0190598. doi: 10.1371/journal.pone.0190598 (PMC5764305; doi:10.1371/journal.pone.0190598)
Supplement: S1 Fig — The full plots from which Fig 3 is derived. (PDF) [file pone.0190598.s004.pdf]

Biplots

The biplots produced by the LC-BML model, and constructed based on these bilinear decompositions, are shown in Figures S4.1 and S4.2. These are the figures on which the simplified Figure 3 are based. The positions of the value items vary across all values segments, but the locations of the response categories remain the same.

The magnitude of the effect of each item on the probability to endorse a specific rating category is determined by the inner product (also known as the scalar or dot product) between the position of the item in that segment and the vector representing the rating. This can be determined by multiplying the length of the projection of the item point onto the rating vector by the length of the rating vector. Simply put, items which have large positive projections are associated with large probabilities of endorsing that rating, and vice versa. For example, in Segment I the item PO1 has a very large positive projection on rating category 6 (“Not like me at all”), which implies that in Segment I people do not value this item highly. In contrast, item UN1 has a large positive projection on rating category 1 (“Very much like me”), implying that this segment values this aspect of universalism most highly.

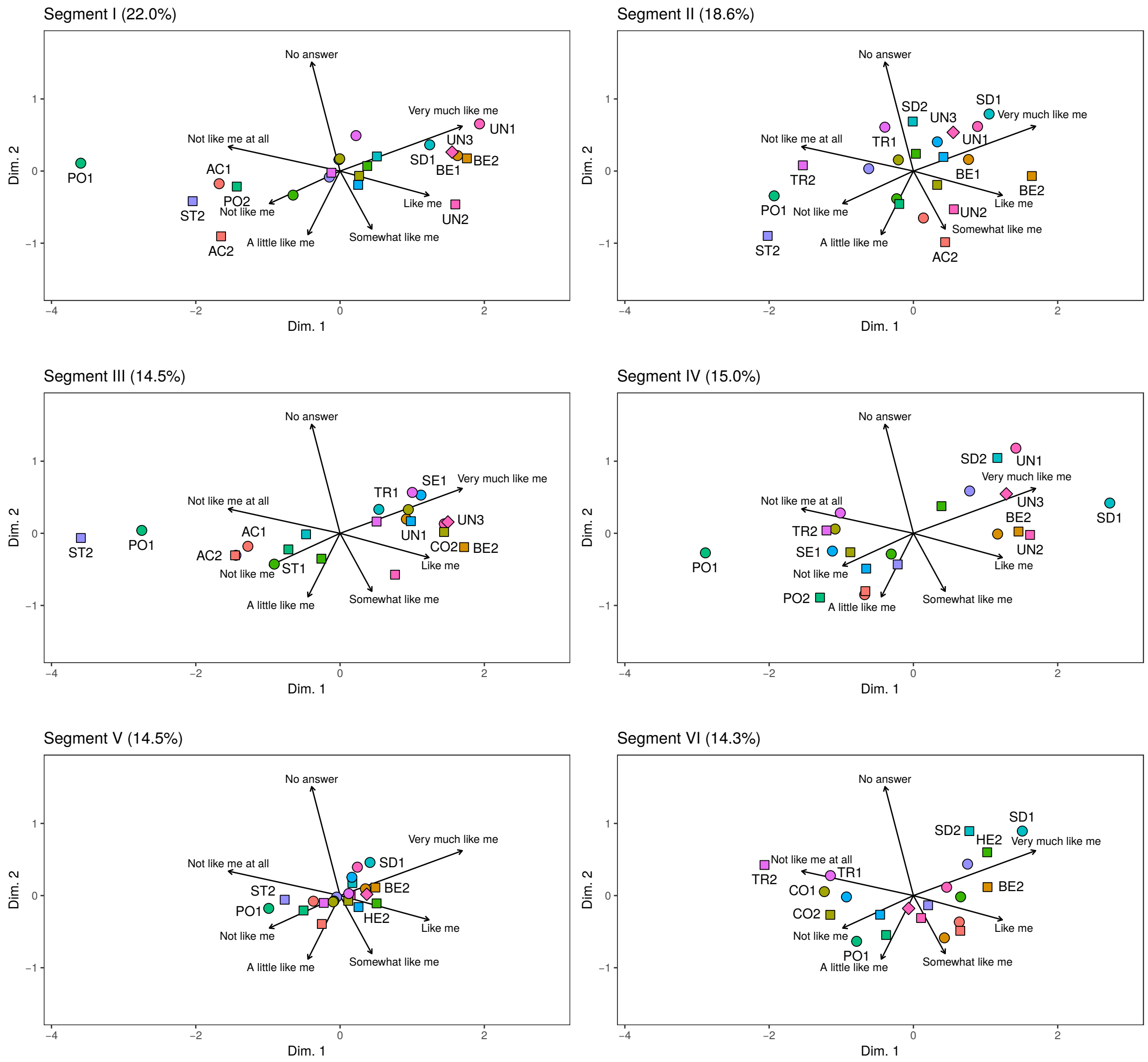

Figure S4.1: Biplots for values segments I – VI. The colours and symbols are the same for all plots, and is explained in the legends of Figure S4.2.

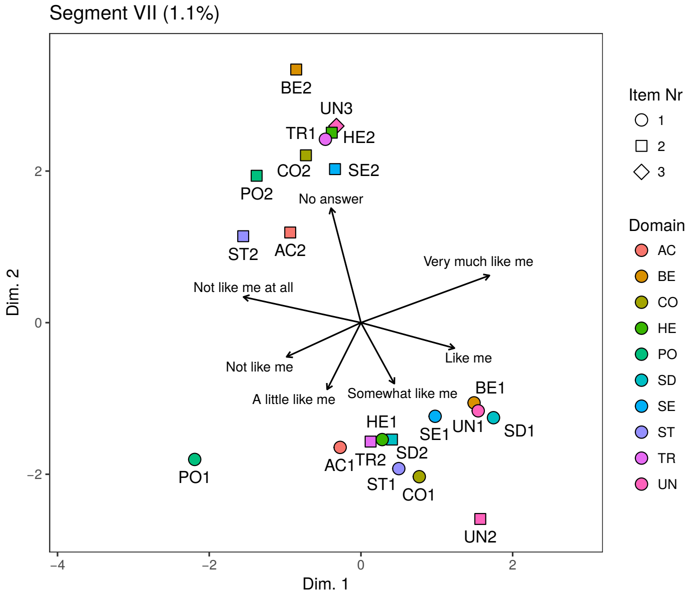

Figure S4.2: Biplot for values segment VII. The legends displayed here also apply to the panels in Figure S4.1. Different colours are used for different values in Schwartz' values system, and the symbol corresponds to the number of the item used to measure that value.
