# Supplementary material for: Competing for the same value segments? Insight into the volatile Dutch political landscape
Source: PLoS One. 2018 Jan 11;13(1):e0190598. doi: 10.1371/journal.pone.0190598 (PMC5764305; doi:10.1371/journal.pone.0190598)
Supplement: S2 File — A discussion of the relationship between socio-demographics and the value segments, and time trends. (PDF) [file pone.0190598.s005.pdf]

Values segments, socio-demographics and stability over time

To assess whether segments differ on socio-demographic characteristics, we calculated distributions of age, education and gender for the respective segments. These distributions are calculated by summing the post-stratification weights of all respondents who fall in a specific category (e.g., all males). Segment membership is incorporated by weighting the post-stratification weights with the estimates of each respondent's posterior segment-membership probabilities in Eq. (5). Table S5.1 shows the distribution of the socio-demographic variables across the segments. The segments differ with respect to socio-demographic characteristics. Segment I contains more females (61.1%) and fewer people younger than 34 (18.6% versus 31.1% in the population). Segment II does not differ much from the average in the population. Segment III differs from the average, it comprises relatively more people who are lower educated (40.6%), female (56.3%), and aged above 60 (42.0%). Segment VI distinguishes itself by containing many highly educated (38.1% versus 24.5% in the population) and people in the middle age category 35 to 59 (52.2%). As Segment III, segment V is low educated, but in contrast to segment III there are people in all age categories and relatively more males (53.4%). In Segment VI, the majority is highly educated (33.3%) young (62.0%) and male (63.3%). These differences in sociodemographics are in line with relationships between values and sociodemographics found in the literature (Schwartz and Rubel, 2009).

As shown in Table S5.2, the sizes of the value segments are relatively stable over time. This is expected as values at a higher aggregation level hardly change [1]. Segment IV has grown somewhat over the years from 12.8% to 16.3%, whilst Segments III and V have decreased in size from 16.1% and 16.0% to 12.3% and 13.2% respectively. Overall, Segment VII is estimated to contain only 1.1% of the population, while the other segments range in size from 14.3% to 22.0%. Segment VII consisting of people who had many missing values (no answer) on a subset of the items seems stable across waves of the ESS.

	**Gender**		**Education Years**			**Age**			
**Segment**	**Male**	**Female**	**1–10**	**11–15**	**16–30**	**15–34**	**35–59**	**60–96**	**Size**
I	38.9	61.1	28.4	50.1	21.5	18.6	52.0	29.5	22.0
II	52.4	47.6	22.2	48.9	28.8	30.2	50.0	19.8	18.6
III	43.7	56.3	40.6	44.7	14.6	12.8	45.3	42.0	14.5
IV	49.3	50.7	14.0	47.9	38.1	34.2	52.2	13.6	15.0
V	53.4	46.6	35.2	46.6	18.3	33.9	41.7	24.4	14.5
VI	63.3	36.7	15.1	51.6	33.3	62.0	33.6	4.4	14.3
VII	42.7	57.3	29.7	53.9	16.4	26.3	41.1	32.6	1.1
Average	49.1	50.9	26.5	49.1	24.5	31.1	45.1	23.8	100.0

Table S5.1: The distribution of the socio-demographic variables across value segments, and the segment sizes. All values are expressed as percentages per row and variable, except for the segment sizes. Unadjusted post-stratification weights are applied.

Table S5.2: **Values segment sizes per year.**

	**ESS Wave**					
**Segment**	**2002**	**2004**	**2006**	**2008**	**2010**	**Overall**
I	21.4	22.2	22.5	21.2	22.7	22.0
II	18.3	18.8	18.2	18.5	19.3	18.6
III	16.1	15.2	13.6	14.9	12.3	14.5
IV	12.8	13.1	16.8	16.8	16.3	15.0
V	16.0	15.7	13.2	14.1	13.2	14.5
VI	14.3	13.9	15.0	13.4	14.7	14.3
VII	1.1	1.1	0.8	1.1	1.6	1.1

Values segment sizes per year (in percentage), using unadjusted post-stratification weights. Each column sums to 100.

References

[1] Schwartz, Shalom H, 2006. "A Theory of Cultural Value Orientations: Explication and Applications." *Comparative Sociology* 5:137–182.
