# Supplementary material for: Competing for the same value segments? Insight into the volatile Dutch political landscape
Source: PLoS One. 2018 Jan 11;13(1):e0190598. doi: 10.1371/journal.pone.0190598 (PMC5764305; doi:10.1371/journal.pone.0190598)
Supplement: S2 Fig — Similar to Fig 4, but with all the ESS waves displayed in a single plot. (PDF) [file pone.0190598.s006.pdf]

Correspondence Analysis of Values Segments and Voting Behaviour

Figure S6.1 shows a version of Figure 4 where all the years are presented in a single plot. The points indicating the same political party are connected by lines.

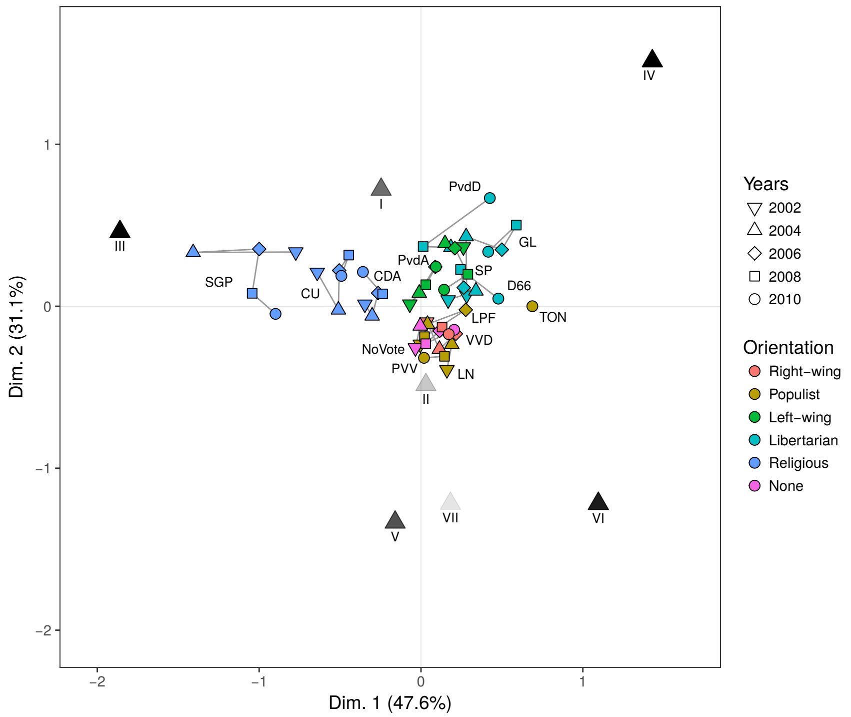

Figure S6.1: Correspondence analysis for the segments and reported votes, using the adjusted poststratification weights applied to the posterior probabilities. The symbols for the segments are faded according to the explained inertia so that darker points fit better. No shading was done for the political parties.
